# Phenotypic diversity and morphometric patterns of Creole goats in the Peruvian Amazonian: A multivariate approach

**DOI:** 10.14202/vetworld.2026.1850-1866

**Published:** 2026-05-10

**Authors:** Aníbal Rodríguez-Vargas, Lucinda Tafur-Gutiérrez, Emmanuel Alexander Sessarego, José Ruiz-Chamorro, Cecilio Barrantes-Campos, Juancarlos Cruz-Luis

**Affiliations:** 1 Dirección de Servicios Estratégicos Agrarios, Instituto Nacional de Innovación Agraria (INIA), Lima 15024, Lima, Perú; 2 Estación Experimental Agraria Amazonas, Instituto Nacional de Innovación Agraria, Utcubamba 01621, Amazonas, Perú; 3 Programa de Investigación y Proyección Social en Animales Menores, Universidad Nacional Agraria La Molina, Lima 15024, Lima, Perú

**Keywords:** animal genetic resources, body indices, Creole goats, morphometric analysis, multivariate analysis, phenotypic characterization, principal component analysis, tropical production systems

## Abstract

**Background and Aim:**

Creole goats (*Capra hircus*) constitute an important genetic resource in tropical production systems; however, their morphostructural characterization in the Peruvian Amazonian remains limited. This study aimed to evaluate the morphological and morphometric characteristics, body indices, and multivariate structure of Creole goats to identify patterns of phenotypic variability and functional adaptation under extensive systems.

**Materials and Methods:**

A total of 149 adult Creole goats were evaluated across three districts (Bagua Grande, Cumba, and El Milagro). 15 qualitative traits and 31 morphometric variables were recorded using standardized protocols. Six ethnological indices; body index (BI), cephalic index (CI), facial index (FI), thoracic index (TI), pelvic index (PI), and proportionality index (PrI), and nine productive indices; thoracic metacarpus index (TMI), costal metacarpus index (CMI), posterior podal index, relative thoracic depth index (RTDI), transverse pelvic index, longitudinal pelvic index, compactness index (CoI), relative cane thickness index, and cane load index, were calculated. Statistical analyses included descriptive statistics, Fisher’s exact test, Z-test, Mann–Whitney U test, Student’s t-test, and Welch’s t-test. Relationships among indices were assessed using Pearson’s correlation coefficient, and multivariate structure was explored using principal component analysis (PCA).

**Results:**

Significant sexual dimorphism was observed, with males showing higher body weight, greater skeletal robustness, and higher FI, TMI, and CoI, whereas females exhibited higher TI and distinct mammary traits. Ecotype differentiation revealed that longilinear goats had elongated conformations with lower PrI values, while brevilinear goats exhibited compact and robust structures with higher TMI, CMI, and cane load index values. Strong correlations were identified between BI and FI, as well as among productive indices such as TMI, RTDI, and CoI (p ≤ 0.001). PCA explained 57.4% of total variability, with body weight and thoracic-related measurements contributing most to the first component, confirming their importance in morphostructural differentiation.

**Conclusion:**

Creole goats in the Peruvian Amazonian exhibit high phenotypic variability and clear morphostructural differentiation influenced by sex, ecotype, and environment. Body measurements and indices provide reliable tools for field-based selection and characterization. The integration of morphometric and multivariate approaches offers a robust framework for genetic improvement, conservation, and sustainable management of goat populations in tropical production systems.

## INTRODUCTION

Creole goats (*Capra hircus*) play an essential role in small ruminant production systems in tropical regions, where their hardiness and adaptability contribute significantly to food security and income generation for smallholder producers operating under environmental and technological constraints [1, 2]. Despite their importance, there is a lack of systematic morphostructural characterization of Creole goat populations in Peru, particularly within tropical ecosystems such as the Amazon. This limitation hampers informed decision-making regarding management practices, conservation strategies, and genetic improvement programs [3].

Phenotypic characterization and the analysis of zoometric indices are fundamental tools for describing body variability, identifying ecotypes, and establishing selection criteria for breeding programs [4–6]. Studies conducted in Asia and Africa have demonstrated the effectiveness of these approaches in identifying morphological patterns and adaptive traits in tropical environments [7, 8]. However, comparable information for Peruvian Creole goats remains scarce and fragmented, especially within Amazonian ecosystems [9].

Despite the recognized importance of Creole goats as resilient genetic resources in tropical production systems, their morphostructural characterization in Peru, particularly within Amazonian ecosystems, remains explored. Existing studies from other regions (e.g., Africa and Asia) have demonstrated the value of morphometric traits and multivariate approaches in identifying adaptive phenotypes and functional ecotypes; however, such systematic, region-specific analyses remain scarce for Peruvian Creole goat populations. Available information is fragmented, lacks standardized methodologies, and often fails to integrate qualitative and quantitative data using advanced statistical tools such as principal component analysis (PCA). Furthermore, there is limited understanding of how microenvironmental variability within Amazonian districts influences phenotypic expression, body indices, and adaptive morphostructural patterns. This gap restricts the development of evidence-based breeding, conservation, and management strategies tailored to local production systems.

In addition, previous studies have rarely combined ethnological and productive indices with multivariate frameworks to establish functional classification criteria applicable under extensive management conditions. The absence of comprehensive morphometric databases for Amazonian goat populations further limits comparative analyses and the identification of selection benchmarks. Consequently, there is a clear need for integrative, field-based research that captures phenotypic diversity, evaluates structural relationships among traits, and links these findings to practical applications in genetic improvement and sustainability.

In response to these gaps, the present study was designed to provide a comprehensive phenotypic and morphometric characterization of Creole goats in the Peruvian Amazonian using a multivariate analytical framework. Specifically, the study aimed to (i) evaluate qualitative morphological traits and quantitative morphometric measurements in adult goats across different districts, (ii) estimate and compare ethnological and productive body indices according to sex, ecotype, and geographic location, and (iii) identify patterns of morphostructural variability and functional adaptation through correlation analysis and PCA.

Furthermore, the study sought to establish a robust morphometric database and generate scientifically grounded criteria for the functional classification of Creole goats under extensive tropical systems. By integrating traditional zoometric evaluation with multivariate statistical approaches, the research aimed to provide practical tools for field-based selection, support genetic improvement programs, and contribute to the conservation and sustainable management of locally adapted goat populations in the Amazonian region.

## MATERIALS AND METHODS

### Ethical approval

This study was conducted in accordance with internationally accepted ethical principles for animal research and welfare applicable to production animals. All procedures involved only non-invasive phenotypic observations and external morphometric measurements performed under routine field management conditions, without the application of experimental treatments, surgical interventions, blood collection, restraint beyond normal handling, or any procedure likely to cause pain, injury, or distress to the animals. Therefore, the study posed minimal risk to animal welfare.

Before data collection began, the study objectives and procedures were clearly explained to all goat owners and farm managers, and verbal informed consent was obtained for the inclusion of their animals and production units in the study. Measurements were performed during routine herd handling to minimize disturbance, and animals were handled calmly and individually by trained personnel to avoid unnecessary stress. Special care was taken to ensure that no animal was forced into prolonged restraint and that all evaluations were completed efficiently under field conditions.

The study adhered to the principles of animal welfare, scientific integrity, confidentiality, and responsible data use. The identities of producers and production units were protected through the anonymization of records, and the collected information was used exclusively for scientific purposes. Because the investigation was based solely on observational and non-invasive procedures carried out during normal management practices, and no biological samples or invasive manipulations were performed, formal approval from an institutional animal ethics committee was not required under the applicable framework for this type of field-based zootechnical study. Nevertheless, the study was performed in full compliance with recognized international recommendations for the ethical use of animals in research and with due respect for animal welfare at every stage of the work.

### Study period and location

**Figure 1:**
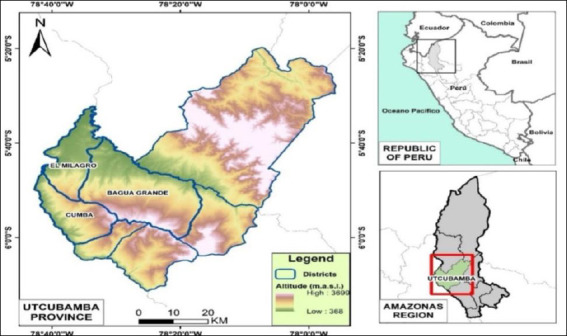
Geographic location of the study area.

The investigation was conducted between June and September 2024 in the districts of Bagua Grande, Cumba, and El Milagro, located in the province of Utcubamba, Amazonas region, Peru (Figure 1). The study area comprises a strategic territory of 3,859.93 km², representing 9.83% of the regional territory. It is bordered by Bagua and Condorcanqui to the north, Bongará to the east, Luya to the south, and Cajamarca to the west. Its geographic diversity and location favor agricultural development, reinforcing its importance in this study. The districts exhibit marked altitudinal variability, ranging from low-lying areas near the Utcubamba River to mountainous regions, with Bagua Grande at 440 meters above sea level (masl), Cumba at 504 masl, and El Milagro at 400 masl. These conditions create heterogeneous environments that influence the phenotypic expression and zoometric indices of the goats. The climate varies according to altitude; in low-lying areas (400–1,400 meters above sea level), a warm climate prevails, with temperatures up to 40°C and annual rainfall close to 1,300 mm, while in higher elevations (1,400–2,900 meters above sea level), a temperate climate predominates, with temperatures between 14°C and 25°C and rainfall ranging from 500 to 3,500 mm per year.

### Study design, sampling strategy, and animal selection criteria

The regional goat population was estimated at 14,827 individuals [10]. A sample of 149 adult Creole goats was collected, including non-pregnant females and males ≥2 years of age, with an average body condition score of 3.0 and age estimated from the number of permanent incisors (2–4). Sampling was conducted using a stratified design that simultaneously considered herd size per production unit, management system (traditional extensive system [SET] and improved extensive system [SEM]), and geographic distribution by district. Under this scheme, the sample was distributed among Bagua Grande (n = 30), Cumba (n = 30), and El Milagro (n = 89), with a larger proportion allocated to the latter district due to its higher goat density and number of herds, ensuring representativeness of the population structure and minimizing spatial bias.

The sample size was calculated using the formula: n = (Z² × p × q × N) / (E² × (N − 1) + Z² × p × q), where n is the sample size, N is the total population, Z is the confidence level (90%), p is the expected proportion, q is its complement, and E is the allowed error (5%). The sampled production units correspond to the predominant type of local goat farming, characterized by extensive management. The SET system corresponds to producers with basic infrastructure and limited health assistance, whereas the SEM system includes larger herds, greater forage support, and more frequent health and reproductive practices [11]. In both systems, animals were maintained under continuous grazing on native vegetation. Health management was empirical, with irregular deworming and no vaccination programs, while reproduction was achieved through natural mating without inbreeding control, thereby preserving traditional genetic stock. These elements representatively reflect the local production context and reduce the risk of selection bias from atypical farms.

### Morphological (qualitative) characterization

**Figure 2:**
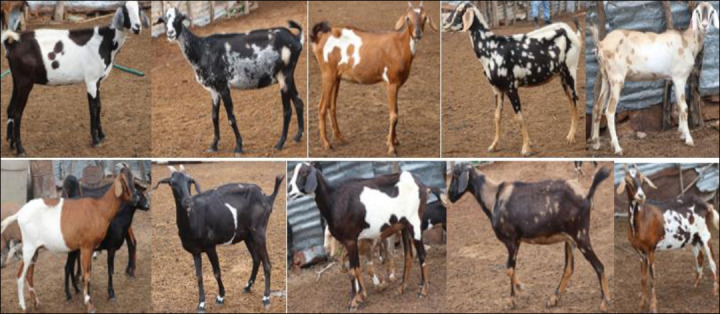
Variation in coat color in evaluated populations of Amazonian Creole goats (2–4 teeth).

Morphological characterization was performed in accordance with international phenotypic evaluation protocols [12–21]. Fifteen qualitative traits were recorded by direct inspection, including head profile, ear size and orientation, coat color and coat length (Figure 2), presence and shape of horns, beard, teats, mucous membrane pigmentation, hoof color, and scrotal bipartition. In females, mammary morphology (udder type and color, and teat direction) was also evaluated, and the body ecotype was classified as brevilinear, mesolinear, or longilinear. Observations were conducted in the morning by a single trained evaluator to minimize animal stress and reduce interobserver variability, and all categories were recorded on previously validated forms for goat phenotyping studies.

### Morphometric (quantitative) measurements

Morphometric measurements were performed following validated methodologies for zoometric studies [22–25]. In each animal, 31 linear variables and perimeters corresponding to the head, neck, trunk, abdomen, rump, limbs, and reproductive structures were recorded. Measurements included body weight; head dimensions; neck length and circumference; chest width and circumference; heights at the withers, back, and rump; body length, width, and depth; abdominal circumference; sternal-dorsal and bicostal diameters; cannon bone and hind bone circumferences; and height at the hock. In males, scrotal length and circumference were measured, whereas in females, mammary morphometry (udder depth, length, and diameter), distance between teats, and individual teat dimensions were recorded.

### Body indices: estimation, correlation analysis, and multivariate analysis

Six ethnological indices, body index (BI), cephalic index (CI), facial index (FI), thoracic index (TI), pelvic index (PI), and proportionality index (PrI), and nine productive indices, thoracic metacarpus index (TMI), costal metacarpus index (MCI), posterior podal index (PPI), relative thoracic depth index (RTDI), transverse pelvic index (TPI), longitudinal pelvic index (LPI), compactness index (CoI), relative cane thickness index (RCTI), and cane load index (CLI), were estimated [26, 27]. These indices were compared by sex, district, and ecotype.

The relationships between ethnological and productive indices were assessed using Pearson’s correlation coefficient. Assumptions of normality and linearity were verified using histograms and scatter plots. Analyses were performed separately for females and males to identify dimorphic patterns. Correlation matrixes and scatter plots were generated to visualize associations among cephalic, body, thoracic, pelvic, and bone robustness dimensions, and correlations were interpreted based on their significance levels (p ≤ 0.05, p ≤ 0.01, p ≤ 0.001).

Additionally, PCA was applied to characterize the multivariate structure of the zoometric measurements. The adequacy of the analysis was verified using the Kaiser–Meyer–Olkin (KMO) index and Bartlett’s test of sphericity. PCA was performed on the correlation matrix of standardized variables, retaining components with eigenvalues > 1, and based on the screen plot. Interpretation was based on factor loadings and communalities, and the selected components were represented using biplots and multivariate projections to visualize the distribution of animals and explore possible groupings by sex, ecotype, or district for descriptive purposes.

### Measurement and quality control protocol

Prior to sampling, the calibration of all instruments was verified: tapes and rulers were checked against a metric standard, calipers were adjusted with standard gauges, and scales were validated with certified weights, ensuring instrumental accuracy and traceability of the records. Measurements were performed in the morning following standardized zoometric protocols by a single trained operator using a flexible measuring tape, a calibrated zoometric stick, a caliper, and a portable digital scale to ensure consistency and minimize variability.

### Statistical analysis

Qualitative variables were analyzed using percentage frequencies. Sexual dimorphism was assessed using Fisher’s exact test, while comparisons of proportions between sexes were performed using the Z-test for two independent proportions. Quantitative variables were expressed as mean ± standard error. Prior to comparisons, normality was assessed using the Shapiro–Wilk test, and homogeneity of variances was assessed using Levene’s test. When normality was not met, the Mann–Whitney U test was applied; for normally distributed data with homogeneous variances, Student’s t-test was used; and in cases of heteroscedasticity, Welch’s t-test was applied due to its robustness against unequal variances.

Statistical significance was interpreted at p ≤ 0.05, p ≤ 0.01, and p ≤ 0.001 under a 95% confidence level. Linear associations between ethnological and productive indices were evaluated using Pearson’s correlation coefficient, estimated by sex for exploratory purposes and for the development of predictive models of weight and other productive characteristics in Creole goats [28]. Comparisons of biometric indices between districts and ecotypes were performed using one-way analysis of variance, followed by Duncan’s post hoc test when assumptions were met; otherwise, the Kruskal–Wallis test and Dunn’s post hoc test were applied. Bonferroni correction was used for multiple comparisons to control for Type I error. PCA was also used to reduce dimensionality and explore multivariate structure, with factor loadings ≥ 0.40 and communalities ≥ 0.50 considered for interpretation. All analyses were performed using R v.4.3.1 software [29].

## RESULTS

### Morphological characterization in goats

Table 1 shows a high frequency of straight cephalic profiles in both sexes (94.7% in females; 94.4% in males; p = 0.2870), indicating homogeneity. Medium-sized ears (75.2 and 69.4%) and horizontal orientation (56.6 and 50.0%) predominated, with no significant differences (p > 0.05). Differences were found in the presence of horns (p = 0.0345), with a higher proportion of dehorned males (63.9%). The shape of the horns also differed (p = 0.0283), with a higher frequency of lyre-shaped horns in females (54.9%). The beard was more common in males (80.6%; p < 0.001). There were no differences in mammae (p = 0.3367), coat color (p = 0.5872), mucous membranes (p = 0.8489), or hooves (p = 1.0000), indicating phenotypic stability.

**Table 1:** Distribution of categorical variables associated with morphological characteristics in Creole goats, according to sex and analysis of statistical significance (p-value).

Variable	Category	Total	Female (n = 113)	Male (n = 36)	p-value
Head profile	Concave	7	6 (5.3%)	1 (2.8%)	0.2870
Convex	1	0 (0.0%)	1 (2.8%)	--	--
Straight	141	107 (94.7%)	34 (94.4%)	--	--
Ear size	Small	2	2 (1.8%)	0 (0%)	0.6479
Mediums	110	85 (75.2%)	25 (69.4%)	--	--
Big	37	26 (23.0%)	11 (30.6%)	--	--
Direction of ears	Horizontal	82	64 (56.6%)	18 (50%)	0.5650
Pendulums	67	49 (43.4%)	18 (50%)	--	--
Horns	Absence	71	48 (42.5%)	23 (63.9%)	0.0345*
Presence	78	65 (57.5%)	13 (36.1%)	--	--
Horn shape	Curved	5	3 (2.6%)	2 (5.5%)	0.0283*
Dehorned	71	48 (42.5%)	23 (63.9%)	--	--
Lyre-shaped	73	62 (54.9%)	11 (30.6%)	--	--
Beard	Absence	89	82 (72.6%)	7 (19.4%)	<0.0000***
Presence	60	31 (27.4%)	29 (80.6%)	--	--
Breasts	Absence	144	108 (95.6%)	36 (100%)	0.3367
Presence	5	5 (4.4%)	0 (0%)	--	--
Coat color	Bay	6	5 (4.3%)	1 (2.8%)	0.5872
White	7	7 (6.2%)	0 (0.0%)	--	--
White and its variants	75	57 (50.6%)	18 (50.0%)	--	--
Brown	14	10 (8.8%)	4 (11.1%)	--	--
Black	9	6 (5.3%)	3 (8.3%)	--	--
Black and its variants	20	14 (12.4%)	6 (16.6%)	--	--
Lead	4	2 (1.8%)	2 (5.6%)	--	--
Roano	14	12 (10.6%)	2 (5.6%)	--	--
Mucous membranes	Pigmented	85	65 (57.5%)	20.0 (55.6%)	0.8489
Not Pigmented	64	48 (42.5%)	16.0 (44.4%)		
Hoof color	White	10	8 (7.1%)	2.0 (5.6%)	1.0000
Black	139	105 (92.9%)	34.0 (94.4%)		
Ecotype (BI)	Brevilineal	6	4 (3.5%)	2 (5.6%)	<0.0000***
Mesolineal	96	93 (14.2%)	3 (8.3%)	--	--
Longilineo	47	16 (82.3%)	31 (86.1%)	--	--
Scrotal bipartition	Absence	14	--	14.0 (38.9%)	0.0594
Presence	22	--	22.0 (61.1%)		
Udder-shaped	Bagged	56	56 (49.6%)	--	<0.0000***
Globose	40	40 (35.4%)	--	--	--
Toothpick	17	17 (15.0%)	--	--	--
Udder-color	White	5	5 (4.4%)	--	<0.0000***
Red	9	9 (8.0%)	--	--	--
Brown	1	1 (0.8%)	--	--	--
Black	89	89 (78.8%)	--	--	--
Pink	9	9 (8.0%)	--	--	--
Direction-nipples	Divergent	96	96 (85%)	--	<0.0000***
Parallels	17	17 (15%)	--	--	--

BI = Body mass index, significant (*p ≤ 0.05), highly significant (**p ≤ 0.01), very highly significant (***p ≤ 0.001), using Z-test.

The body ecotype showed highly significant differences (p < 0.001), with a predominance of the longilinear biotype in males (86.1%). Scrotal bipartition tended to be significant (p = 0.0594), with a higher frequency of presence (61.1%). In females, highly significant differences were observed in mammary morphology: baggy udder (49.6%; p < 0.001), black color (78.8%), and divergent nipples (85%; p < 0.001), traits associated with greater breastfeeding efficiency.

### Morphometric measurements in goats

Table 2 shows significant differences between sexes in several morphometric variables. Males presented higher values for body weight (47.94 vs. 39.66 kg), head length and width, neck circumference, and chest width (p < 0.001). They also recorded higher values for height at the withers, height at the base of the tail, and sternodorsal diameter (p < 0.05), as well as greater cannon bone circumference and height at the hock (p < 0.001).

**Table 2:** Distribution of zoometric variables in goats by sex and analysis of statistical significance (p-value).

Variable	Unit of measurement	Female (n = 113)		Male (n = 36)		p-value

		Average	IS	Average	IS	
BOW	kg	39.66^b^	0.87	47.94 ^a^	2.21	0.0002***
HEL	cm	22.47 ^b^	0.21	23.96 ^a^	0.37	0.0002***
FAL	cm	15.19 ^a^	0.14	15.39 ^a^	0.30	0.7098
HEW	cm	13.89 ^b^	0.11	14.72 ^a^	0.26	0.0003***
EAL	cm	18.18 ^a^	0.24	19.00 ^a^	0.46	0.0964
NEL	cm	30.53 ^a^	0.37	30.90 ^a^	0.42	0.3889
NEG#	cm	32.51 ^b^	0.35	40.86 ^a^	1.15	<0.0000***
CHW	cm	19.66 ^b^	0.27	22.04 ^a^	0.49	<0.0000***
THG#	cm	81.16 ^a^	0.57	83.46 ^a^	1.52	0.1628
WIH	cm	69.00 ^b^	0.51	70.24 ^a^	1.75	0.0426*
BAH	cm	69.37 ^a^	0.62	71.47 ^a^	0.92	0.0615
RUH#	cm	72.13 ^b^	0.44	74.38 ^a^	1.06	0.0570
BTH	cm	61.81 ^b^	0.38	64.75 ^a^	1.01	0.0018**
RUW	cm	16.99 ^a^	0.20	17.38 ^a^	0.40	0.1529
RUL	cm	20.36 ^a^	0.18	21.00 ^a^	0.40	0.1060
BOWL	cm	77.55 ^a^	0.65	79.65 ^a^	1.52	0.2051
BOD	cm	33.20 ^a^	0.45	32.68 ^a^	0.69	0.9823
ABG#	cm	91.46 ^a^	0.77	92.26 ^a^	1.81	0.6844
DSD#	cm	29.23 ^b^	0.23	31.28 ^a^	0.54	0.0010**
IDB	cm	26.31 ^a^	0.28	26.03 ^a^	0.59	0.6372
FCG	cm	8.80 ^b^	0.08	10.00 ^a^	0.20	<0.0000***
RCG	cm	9.40 ^b^	0.08	10.68 ^a^	0.19	<0.0000***
HOH	cm	27.95 ^b^	0.27	30.06 ^a^	0.34	<0.0000***
SCC	cm	--	--	28.75	0.42	--
SCL	cm	--	--	16.36	0.51	--
UDD	cm	13.06	0.26	--	--	--
UDL	cm	15.7	0.27	--	--	--
UDI	cm	30.32	0.50	--	--	--
DIB	cm	9.20	0.21	--	--	--
NID	cm	7.65	0.23	--	--	--
NIL	cm	7.02	0.21	--	--	--

a, b: Means within the same row with different superscripts represent significant differences (p < 0.05); BOW: Body weight, HEL: Head length, FAL: Face length, HEW: Head width, EAL: Ear length, NEL: Neck length, NEG: Neck circumference, CHW: Chest width, THG: Thoracic circumference, WIH: Height at the withers, BAH: Back height, RUH: Croup height, BTH: Height at the base of the tail, RUW: Croup width, RUL: Croup length, BOL: Body length, BOD: Body depth, ABG: Abdominal circumference, DSD: Sternodorsal diameter, BID: Bicostal diameter, FCG: Fore-cannon bone circumference, RCG: Hind-cannon bone circumference, HOH: Height at the hock, SCC: Scrotal circumference, SCL: Scrotal length, UDD: Udder depth, UDL: Udder length, UDI: Udder diameter, DIB: Distance between teats, NID: Teat diameter, NIL: Teat length, SE: Standard errors; significant (*p ≤ 0.05); highly significant (**p ≤ 0.01); very highly significant (***p ≤ 0.001); (#) Welch’s t-test. Variables marked with (#) were analyzed using Welch’s t-test for normal distribution and heterogeneous variances; the remaining variables were analyzed using the Mann–Whitney U test.

No significant differences were observed between sexes in face length, ear length, neck length, chest circumference, rump dimensions, body length and depth, or abdominal circumference (p > 0.05) (Table 3). For reproductive variables, males had defined values for scrotal circumference and length, while females had udder measurements of anterior udder diameter (30.32 cm) and udder depth (13.06 cm).

**Table 3:** Assessment of body index according to ethnological and productive interest by sex in Creole goats.

Category	Variable	Female (n = 113)	SE	Male (n = 36)	SE	p-value
Ethnological Interest	BI	95.64^a^	0.58	95.50^a^	0.77	0.6703
	CI	62.25^a^	0.61	61.70^a^	1.02	0.6224
	FI	91.98^b^	0.90	96.42^a^	1.77	0.0045**
	YOU	90.23^a^	0.87	83.43^b^	1.62	0.0004***
	PI#	83.43^a^	0.65	82.68^a^	1.10	0.5710
	PrI	89.24^a^	0.57	88.80^a^	2.22	0.5854
Productive Interest	MTI#	10.86^b^	0.09	12.03^a^	0.20	<0.0000***
	MCI	33.80^b^	0.43	38.85^a^	0.88	<0.0000***
	PPI	45.27^a^	0.42	46.66^a^	0.60	0.0673
	RTDI	42.48^b^	0.32	46.72^a^	2.98	0.2364
	TPI	24.64^a^	0.24	25.96^a^	1.82	0.6272
	LPI#	28.23^a^	0.18	28.26^a^	0.42	0.9355
	CoI	57.17^b^	0.99	71.20^a^	5.93	0.0005***
	RCTI	12.78^b^	0.11	14.86^a^	0.89	<0.0000***
	CLI	23.00^a^	0.39	22.09^a^	0.86	0.2868

a, b: Means within the same row with different superscripts represent significant differences (p < 0.05), BI = Body index, CI = Cephalic index, FI = Facial index, TI = Thoracic index, PI = Pelvic index, PrI = Proportionality index, MTI = Metacarpo-thoracic index, MCI = Metacarpo-costal index, PPI = Posterior foot index, RTDI = Relative thoracic depth index, TPI: Transverse pelvic index, LPI = Longitudinal pelvic index, CoI = Compactness index, RCTI = Relative cannon thickness index, CLI = Cannon load index, significant (*p ≤ 0.05), highly significant (**p ≤ 0.01), very highly significant (***p ≤ 0.001), # Independent t-test. Variables marked with “#” were analyzed using an independent-samples t-test, as they showed normal distributions and homogeneous variances.

Table 4 presents the body indices of goats from Bagua Grande, Cumba and El Milagro. Among the ethnological indices, BI showed significantly higher values in Bagua Grande and El Milagro compared to Cumba (p < 0.05). FI was also significantly higher in El Milagro, followed by Bagua Grande (p < 0.05). No significant differences were detected between districts in CI, TI, PI, or PrI (p > 0.05).

**Table 4:** Assessment of body index according to ethnological and productive interest by district in Creole goats.

Category	Variable	Bagua Grande (n = 30)	Median	Cumba (n = 30)	Median	El Milagro (n = 89)	Median	p-value
Ethnological Interest	BI	96.26 ± 1.29^a^	96.84	93.51 ± 1.07^b^	93.66	96.10 ± 0.55^ab^	96.05	0.0850
	CI#	62.73 ± 1.25	63.34^a^	59.86 ± 1.19	59.55^a^	62.67 ± 0.64	62.50^a^	0.1348
	FI	92.89 ± 1.84^ab^	92.86	89.38 ± 1.90^b^	89.12	94.34 ± 1.02^a^	94.12	0.0622
	YOU#	86.75 ± 1.63	88.90^a^	89.52 ± 1.49	91.75^a^	88.90 ± 1.12	89.83^a^	0.3938
	PI	83.61 ± 1.11^a^	84.55	82.65 ± 1.24^a^	81.53	83.33 ± 0.76^a^	83.33	0.8538
	PrI#	89.26 ± 1.23	89.67^a^	90.95 ± 1.10	89.76^a^	88.49 ± 1.00	88.00^a^	0.3498
Productive Interest	MTI#	11.02 ± 0.19	10.74^a^	11.23 ± 0.24	11.11^a^	11.16 ± 0.12	10.97^a^	0.7618
	MCI#	34.59 ± 0.70	33.67^a^	35.46 ± 0.95	34.62^a^	35.01 ± 0.60	34.00^a^	0.7119
	PPI#	46.93 ± 0.80	46.77^a^	45.33 ± 0.94	45.93^a^	45.26 ± 0.41	45.71^a^	0.2750
	RTDI#	43.37 ± 0.56	43.13^a^	42.19 ± 0.59	41.85^a^	44.00 ± 1.25	42.11^a^	0.2222
	TPI#	25.01 ± 0.45	25.36^a^	23.71 ± 0.45	24.09^b^	25.36 ± 0.76	24.67^ab^	0.1032
	LPI	28.41 ± 0.39^a^	28.47	27.78 ± 0.42^a^	28.00	28.34 ± 0.21^a^	28.05	0.3910
	CoI#	54.99 ± 1.84	54.10^b^	59.09 ± 2.18	55.74^ab^	62.93 ± 2.61	59.72^a^	0.1044
	RCTI#	12.88 ± 0.19	12.77^a^	13.22 ± 0.22	13.33^a^	13.44 ± 0.39	13.04^a^	0.5062
	CLI#	24.06 ± 0.76^a^	23.96^a^	23.04 ± 0.74	23.33^ab^	22.26 ± 0.49	22.22^b^	0.1186

a, b: Means within the same row with different superscripts represent significant differences (p < 0.05), BI = Body index, CI = Cephalic index, FI = Facial index, TI = Thoracic index, PI = Pelvic index, PrI = Proportionality index, MTI = Metacarpo-thoracic index, MCI = Metacarpo-costal index, PPI = Posterior foot index, RTDI = Relative thoracic depth index, TPI: Transverse pelvic index, LPI = Longitudinal pelvic index, CoI = Compactness index, RCTI = Relative cannon thickness index, CLI = Cannon load index, #KW and Dunn test. The Kruskal–Wallis test and Dunn’s post hoc test were applied because the residuals did not follow a normal distribution.

In terms of productivity indices, TPI showed significantly higher values in Bagua Grande and El Milagro (p < 0.05). CoI was significantly higher in El Milagro (p < 0.05), whereas CLI was higher in Bagua Grande (p < 0.05).

Table 5 compares body indices among the brevilinear, mesolinear, and longilinear ecotypes. Among the ethnological indices, BI and PrI showed significantly higher values in the longilinear ecotype (p < 0.001). No significant differences were observed between ecotypes in CI, FI, TI, or PI (p > 0.05).

**Table 5:** Assessment of body index according to ethnological and productive criteria, classified by goat ecotype.

Category	Variable	Brevilenio	Median	Mesolineal	Median	Longilineo	Median	p-value
Ethnological Interest	BI#	82.54 ± 0.61	82.41^b^	88.59 ± 0.29	88.89^b^	97.32 ± 0.42	96.59^a^	<0.0000***
	CI#	62.83 ± 2.31	64.61^a^	61.93 ± 1.58	61.90^a^	62.11 ± 0.57	61.95^a^	0.7631
	FI	94.52 ± 4.08^a^	95.33	91.84 ± 2.84^a^	93.33	93.17 ± 0.87^a^	93.33	0.8106
	YOU#	93.38 ± 3.23	90.94^a^	89.96 ± 2.01	90.91^a^	88.15 ± 0.90	89.66^a^	0.5514
	PI	87.37 ± 2.18^a^	88.89	85.13 ± 1.59^a^	85.00	82.76 ± 0.61^a^	81.82	0.1195
	PrI#	96.44 ± 0.81	96.55^a^	95.14 ± 1.71	94.44^a^	87.86 ± 0.73	87.80^b^	<0.0000***
Productive Interest	MTI#	10.02 ± 0.27	9.78^b^	10.82 ± 0.22	10.84^ab^	11.25 ± 0.10	11.11^a^	0.0076**
	MCI#	33.39 ± 1.30	32.33^a^	34.45 ± 0.88	33.33^a^	35.18 ± 0.49	34.70^a^	0.7141
	PPI#	50.20 ± 1.93	48.76^a^	47.44 ± 0.60	48.28^a^	45.11 ± 0.39	45.57^b^	0.0028**
	RTDI#	40.64 ± 0.94	40.98^a^	41.94 ± 0.79	41.43^a^	43.89 ± 0.91	42.59^a^	0.1438
	TPI#	24.64 ± 0.22	24.66^a^	24.60 ± 0.75	25.37^a^	25.03 ± 0.56	24.64^a^	0.8994
	LPI	26.85 ± 0.96^a^	27.00	27.43 ± 0.42^a^	27.40	28.43 ± 0.18^a^	28.47	0.0343*
	CoI#	62.32 ± 4.67	58.09^a^	55.66 ± 1.76	53.97^a^	61.22 ± 1.98	57.13^a^	0.3754
	RCTI#	12.60 ± 0.38	12.06^a^	12.86 ± 0.19	13.04^a^	13.38 ± 0.28	13.14^a^	0.4991
	CLI#	20.66 ± 1.25	22.22^a^	23.45 ± 0.74	23.68^a^	22.78 ± 0.42	22.79^a^	0.3082

a, b: Means within the same row with different superscripts represent significant differences (p < 0.05); BI = Body index, CI = Cephalic index, FI = Facial index, TI = Thoracic index, PI = Pelvic index, PrI = Proportionality index, MTI = Metacarpo-thoracic index, MCI = Metacarpo-costal index, PPI = Posterior foot index, RTDI = Relative thoracic depth index, TPI: Transverse pelvic index, LPI = Longitudinal pelvic index, CoI = Compactness index, RCTI = Relative cannon thickness index, CLI = Cannon load index, significant (*p ≤ 0.05); highly significant (**p ≤ 0.01); very highly significant (***p ≤ 0.001); #KW and Dunn test. The Kruskal–Wallis test and Dunn’s post hoc test were applied because the residuals did not follow a normal distribution.

In terms of productivity indices, MTI was significantly higher in the longilinear ecotype (p < 0.01), whereas PPI was significantly lower in the same ecotype (p < 0.01). LPI showed a significant increase from the brevilinear to the longilinear ecotype (p < 0.05). No significant differences were detected between ecotypes in CLI, RCTI, or CoI (p > 0.05).

### Analysis of the correlation of body indices according to ethnological and productive interest in Creole goats

**Figure 3:**
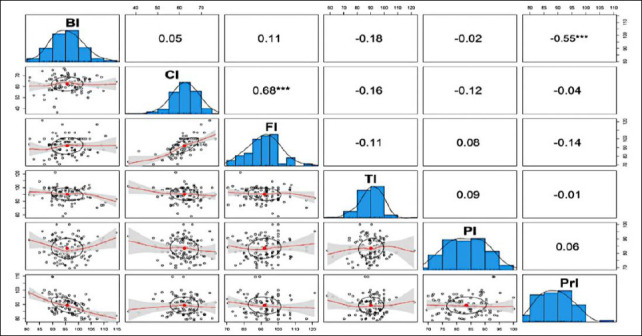
Correlation coefficient between body indices according to ethnological interest in female goats. BI = Body Index, CI = Cephalic Index, FI = Facial Index, TI = Thoracic Index, PI = Pelvic Index, PrI = Proportionality Index, very highly significant (***p ≤ 0.001).

In Figure 3, a strong positive correlation was found between BI and FI (r = 0.68; p ≤ 0.001). A significant negative correlation was also observed between BI and PrI (r = −0.55; p ≤ 0.001). The remaining correlations between the ethnological indices showed low or non-significant coefficients. The scatter plots showed weak linear relationships, and the frequency distributions exhibited approximately normal behavior, supporting the use of Pearson’s correlation coefficient.

**Figure 4:**
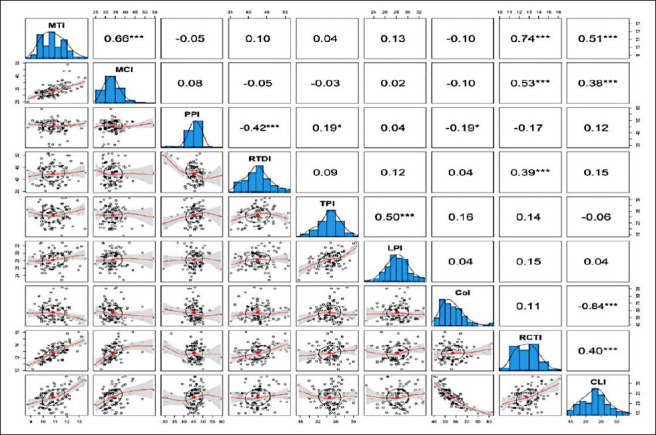
Correlation coefficient between body indices according to productive interest in female goats. TMI = Thoracic Metacarpo-index, MCI = Costal Metacarpo-index, PPI = Posterior Pedal Index, RTDI = Relative Thoracic Depth Index, TPI = Transverse Pelvic Index, LPI = Longitudinal Pelvic Index, CoI = Compactness Index, RCTI = Relative Cannon Thickness Index, CLI = Cannon Load Index, significant (*p ≤ 0.05), highly significant (**p ≤ 0.01), very highly significant (***p ≤ 0.001).

In Figure 4, TMI showed significant positive correlations with MCI (r = 0.66), RCTI (r = 0.74), and CLI (r = 0.51) (p ≤ 0.001). TPI showed a positive correlation with RCTI (r = 0.39; p ≤ 0.001). Significant negative correlations were identified between PPI and RTDI (r = −0.42), as well as between CoI and CLI (r = −0.84) (p ≤ 0.001). Linear patterns and normality of distributions support the statistical validity of the results.

**Figure 5:**
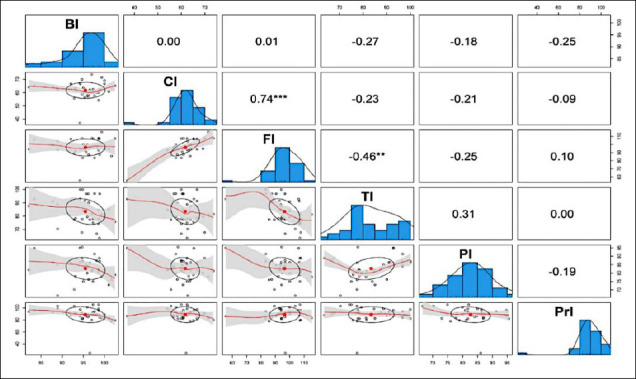
Correlation coefficient between body indices according to productive interest in female goats. TMI = Thoracic Metacarpo-index, MCI = Costal Metacarpo-index, PPI = Posterior Pedal Index, RTDI = Relative Thoracic Depth Index, TPI = Transverse Pelvic Index, LPI = Longitudinal Pelvic Index, CoI = Compactness Index, RCTI = Relative Cannon Thickness Index, CLI = Cannon Load Index, significant (*p ≤ 0.05), highly significant (**p ≤ 0.01), very highly significant (***p ≤ 0.001).

In Figure 5, a strong positive correlation was found between CI and FI (r = 0.74; p ≤ 0.001). A moderate negative correlation was also observed between FI and TI (r = −0.46; p ≤ 0.01). The remaining correlations between the analyzed indices showed low or non-significant coefficients. The histograms showed slightly skewed distributions, particularly for PrI. The scatter plots revealed linear relationships for the significant correlations and random patterns for the remaining associations, supporting the use of a linear correlation approach in the analysis.

**Figure 6:**
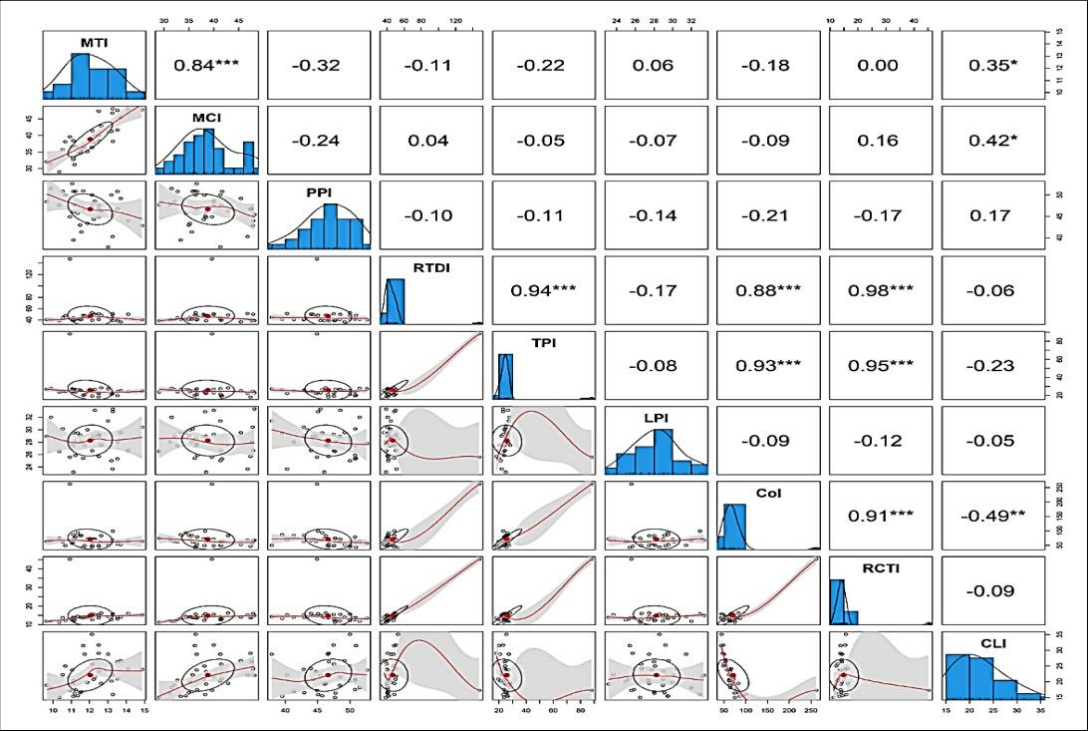
Correlation coefficient between body indices according to productive interest in male goats. TMI = Thoracic Metacarpo-index, MCI = Costal Metacarpo-index, PPI = Posterior Pedal Index, RTDI = Relative Thoracic Depth Index, TPI = Transverse Pelvic Index, LPI = Longitudinal Pelvic Index, CoI = Compactness Index, RCTI = Relative Cannon Thickness Index, CLI = Cannon Load Index, significant (*p ≤ 0.05), highly significant (**p ≤ 0.01), very highly significant (***p ≤ 0.001).

Figure 6 shows a strong positive correlation between TMI and RTDI (r = 0.84; p ≤ 0.001). Similarly, RTDI showed positive correlations with TPI (r = 0.88) and CoI (r = 0.91) (p ≤ 0.001). Likewise, TPI was closely correlated with CoI (r = 0.93) and with RTDI (r = 0.88).

Weaker positive correlations were observed between MCI and CLI (r = 0.42), as well as between TMI and CLI (r = 0.35) (p ≤ 0.05). In contrast, CoI showed a moderate negative correlation with CLI (r = −0.49; p ≤ 0.01). The remaining correlations were weak or not significant.

### PCA

**Figure 7:**
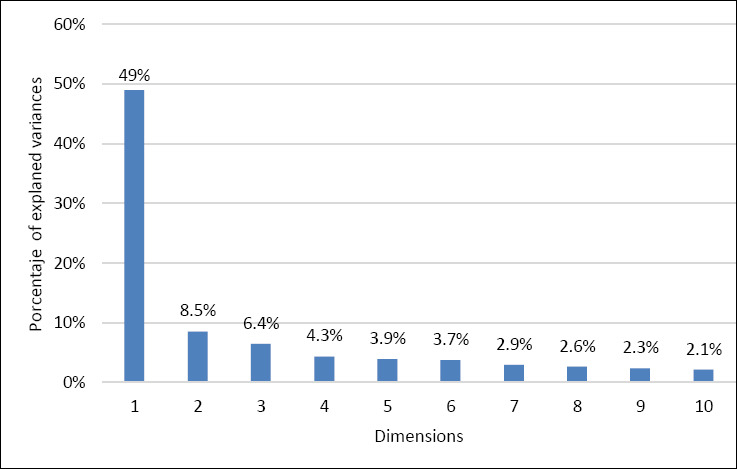
Principal component screen.

Table 6 presents the results of PCA applied to the zoometric measurements in goats, including explained variances and communalities. PCA allowed for dimensionality reduction of the dataset and summarized the observed morphological variation (Figure 7). The first eight components explained more than 80% of the total variance, so the analysis focused on these dimensions.

**Table 6:** Principal component matrix of zoometric measurements in goats, with their respective variance and communality indices.

Variable	CP1	CP2	CP3	CP4	CP5	CP6	CP7	CP8	Communality
Weight	0.2605	–0.0948	0.0055	0.0947	–0.0452	0.0848	–0.0521	0.1879	0.1331
LCA	0.2146	0.0719	–0.0094	0.1041	–0.1399	–0.5603	0.0843	0.2448	0.4627
LC	0.1893	–0.1006	0.1217	0.0072	–0.4208	–0.4754	–0.078	–0.3465	0.5898
HERE	0.1878	–0.1566	–0.2683	–0.0546	0.1994	–0.0182	0.2118	–0.2562	0.2853
IT	0.1318	0.0528	–0.2916	0.5851	0.4221	–0.2774	–0.0059	0.0865	0.7102
LCu	0.1629	0.0586	–0.0177	–0.4675	0.4496	–0.3867	0.1546	–0.0996	0.6343
PCu	0.2296	0.0778	–0.2108	–0.1597	–0.2701	0.0308	0.0903	0.1737	0.2409
AP	0.2349	0.0596	–0.0707	–0.0492	–0.0682	0.1062	0.3461	–0.0087	0.2019
PT	0.2538	–0.1707	0.187	–0.1396	–0.0479	–0.0344	0.0298	0.2066	0.0195
AC	0.202	0.1011	0.1354	–0.1704	0.1673	0.0076	–0.3809	–0.3822	0.4176
AD	0.222	0.108	0.1311	–0.1277	0.1752	0.0505	–0.4274	–0.0784	0.3165
AG	0.2418	0.1811	0.2054	0.1011	0.0732	–0.0118	–0.1335	0.1666	0.1947
ANC	0.2142	0.1473	–0.0663	0.2923	0.1399	0.1476	–0.2602	0.1441	0.2872
ANG	0.2136	0.156	0.2747	0.2006	–0.0239	0.1758	0.4111	–0.2015	0.4267
LG	0.2215	0.1355	0.1893	0.2409	–0.0146	0.1827	0.2697	–0.3968	0.4251
DL	0.254	–0.0804	0.0495	–0.0459	0.1116	0.0835	0.128	0.066	0.1157
PrC	0.0518	–0.5764	0.0269	0.2589	–0.1026	–0.0871	–0.1721	–0.254	0.5149
PA	0.1999	–0.3598	0.178	–0.0483	–0.0403	0.0273	0.0918	0.352	0.3382
DDE	0.2091	–0.1264	–0.2756	–0.2346	0.151	0.1706	0.065	0.0327	0.2479
DB	0.1845	–0.2991	0.3219	–0.0087	0.1248	0.1801	–0.1243	0.104	0.3015
PCA	0.226	0.0083	–0.397	–0.0408	–0.221	0.0843	–0.1269	–0.0841	0.2896
PCP	0.2185	–0.0127	–0.4019	–0.011	–0.2192	0.1973	–0.1624	–0.0792	0.3292
ACO	0.161	0.4619	0.1292	–0.0357	–0.2387	–0.0305	–0.122	0.1087	0.3418
Eigenvalue	11.263	1.9461	1.4728	0.9985	0.8957	0.8549	0.6657	0.6037	–
Variance (%)	48.97	8.46	6.4	4.34	3.9	3.72	2.89	2.63	–
Cumulative Variance (%)	48.97	57.43	63.83	68.17	72.07	75.79	78.68	81.31	–

KMO = 0.92 Bartlett = 1680.934 GL = 253.00 Meaning = 5.79E-21***. LCA = Head length, LC = Face length, ACA = Head width, LO = Ear length, Lcu = Neck length, Pcu = Neck circumference, AP = Chest width, PT = Thoracic circumference, ACA = Height at the withers, AD = Height at the back, AG = Height at the croup, ANC = Height at the tail set, ANG = Croup width, LG = Croup length, DL = Diameter length or body length, PrC = Body depth, PA = Abdominal circumference, DDE = Dorsosternal diameter, DB = Bicostal diameter, PCA = Fore-cannon bone circumference, PCP = Hind-cannon bone circumference, ACO = Height at the hock; significant (*p ≤ 0.05); highly significant (**p ≤ 0.01); very highly significant (***p ≤ 0.001).

**Figure 8:**
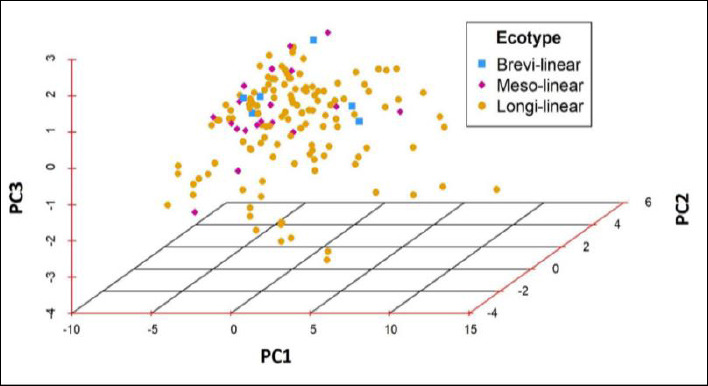
Dispersion of producers by ecotype in the first three main components.

Figure 8 shows the distribution of individuals according to the first three principal components, differentiated by ecotype. A predominance of the longilinear ecotype is observed, with a partially differentiated distribution compared to the other ecotypes and some degree of overlap between groups.

## DISCUSSION

### Morphological characterization and morphometric measurements in Creole goats

The sexual dimorphism observed in Creole goats, expressed primarily in horn presence, beard development, and wattles, is consistent with descriptions in populations adapted to extensive systems and suggests a genetic basis associated with both reproductive functions and environmental adaptation [30–34]. The higher frequency of hornless males with developed beards, compared to females with lyre-shaped horns and limited beard development, has previously been associated with mechanisms of sexual dominance and reproductive and maternal protection strategies [35].

The predominance of a straight cephalic profile in both sexes agrees with findings reported in previous studies [25, 32], although it contrasts with reports describing convex profiles in other goat populations [18]. These discrepancies reinforce the value of the frontonasal profile as an ethnological trait sensitive to genetic variability and processes of racial differentiation in Creole goats [36].

Regarding auricular characteristics, the predominance of medium-sized ears differs from studies reporting large, pendulous ears in other regions [18, 25], which may reflect local adaptations to specific environmental conditions or undirected selection pressures. Similarly, the high variability observed in coat, mucous membrane, and hoof color demonstrates the phenotypic plasticity of Creole goats in response to heterogeneous ecological environments, as reported in African and Latin American populations [37, 38].

Scrotal bipartition, observed in 61.1% of males, exceeded the rates reported in other studies [25, 39]. Previous research suggests that this characteristic may favor testicular thermoregulation in warm environments without compromising fertility when bipartition is partial [40, 41], indicating a potential adaptive advantage under tropical conditions.

In females, the high frequency of pouched udders, black pigmentation, and divergent teats differs from findings that reported a predominance of cylindrical udders [25, 42]. These differences may be associated with functional variations linked to management conditions, environmental factors, and local selection history, given that udder morphology has been linked to milking efficiency and udder health [31].

The morphometric differences between sexes are consistent with patterns widely reported in Creole breeds, in which males exhibit greater body size and more developed secondary sexual characteristics due to reproductive competition and hormonal dimorphism [43, 44]. In contrast, females tend to exhibit conformational traits favoring gestation and lactation, including greater relative thoracic capacity [32]. The higher CoI observed in males reflects increased structural robustness, a trait considered important in selection strategies for extensive production systems [44].

### Assessment of body indices according to ethnological and productive interest in Creole goats

The results demonstrated wide variability in body indices among the evaluated Creole goats, with direct implications for their ethological and productive characterization in extensive systems. Males showed higher values in FI, TMI, and CMI, which are consistent with greater structural robustness and have been associated with increased capacity for support and tolerance of restrictive environmental conditions [45]. In contrast, females exhibited higher TI values, suggesting a conformation associated with greater thoracic functional capacity, a trait relevant for physiological efficiency under grazing conditions [45].

Differences observed between locations highlight the influence of environmental and management conditions on body conformation. In Bagua Grande and El Milagro, higher values of TPI and CoI were recorded, patterns previously associated with improved morphofunctional adaptation in extensive systems [34, 46], whereas lower values were observed in Cumba, consistent with populations exposed to lower levels of structural selection [38].

The ecotype analysis revealed clear contrasts in body conformation. Longilinear individuals exhibited greater body length and lower PrI values, corresponding to a more elongated and lighter structure, which has been described in populations adapted to prolonged movement across extensive areas [47]. In contrast, brevilinear individuals showed higher TMI, CMI, and CLI values, reflecting a more compact and robust conformation associated with greater body mass and structural resistance [48, 49].

CoI and RCTI further reinforced these morphostructural differences. Longilinear ecotypes, characterized by lower CoI values, have been described as more efficient in resource utilization under extensive conditions [14,18], whereas brevilinear ecotypes, with higher RCTI values, exhibit greater bone robustness, a feature associated with animals subjected to higher physical demands [48, 49].

### Analysis of the correlation of body indices according to ethnological and productive interest in Creole goats

Body indices confirmed their usefulness for the phenotypic characterization of Creole goats, consistent with previous findings emphasizing the importance of head proportions in traditional selection processes related to adaptability and hardiness [43, 36]. These morphometric relationships reflect heritable functional patterns that are particularly relevant in extensive production systems.

The inverse relationship between body volume and structural proportionality, previously described [48], is associated with adaptive specialization in climatically restrictive environments. Furthermore, other studies have highlighted the importance of bone and pelvic development for mobility, physical endurance, and reproductive performance, facilitating the identification of phenotypes with greater functional fitness under rustic management conditions [16, 25].

In males, craniofacial proportions play an additional role in ethnic and adaptive differentiation [27, 50], being associated with thermoregulatory mechanisms in arid environments and reinforcing their relevance as selection criteria in pastoral systems. Indices associated with body compactness (CoI) and RTDI showed consistent relationships with functional efficiency in reproductive and load-bearing processes [48, 51]. The observed structural sexual dimorphism is influenced by the differential effects of androgens on body growth [52–54], consistent with previous reports [32, 55].

### PCA

PCA proved to be an effective tool for reducing the dimensionality of morphometric variables and revealing underlying structural patterns in Creole goats. The high proportion of variance explained by the first two components is consistent with previous findings highlighting their usefulness in characterizing local breeds [25, 48]. Differences in accumulated variance percentages may be attributed to genetic, environmental, and methodological factors.

The high factor loadings of thoracic, pelvic, and proportionality-related indices in the initial components confirm their relevance as integrative descriptors of body conformation and their association with meat production potential. These findings support the use of PCA as an objective tool in morphofunctional selection programs, particularly in low-technology production systems [56].

However, the absence of standardized phenotypic selection criteria, combined with transhumance practices and the uncontrolled introduction of specialized breeds, has promoted undirected crossbreeding, increased genetic introgression, and limited the development of populations with homogeneous productive profiles [18].

Overall, PCA provides a synthetic and objective framework for characterizing goat populations with high phenotypic variability and offers a robust analytical basis for guiding selection and conservation strategies in production systems with limited technical and economic resources.

## CONCLUSION

The present study demonstrated clear sexual dimorphism and marked morphostructural variability in Creole goats from the Peruvian Amazonian, reflected in both qualitative traits and quantitative measurements. Males exhibited greater body weight, skeletal robustness, and higher FI, TMI, and CoI values, whereas females showed relatively higher TI values and distinctive mammary characteristics. Ecotype-based differentiation revealed that longilinear animals possess elongated and lighter conformations with lower PrI values, while brevilinear animals exhibit more compact and robust structures with higher TMI, CMI, and CLI values. Correlation analysis confirmed strong functional relationships among indices, particularly between BI and FI, and among productive indices such as TMI, RTDI, and CoI. Furthermore, PCA identified overall body size and thoracic development as the primary sources of variation, explaining a substantial proportion of total variability.

From a practical perspective, these findings highlight the utility of morphometric traits and body indices as reliable, low-cost tools for field-based selection and management under extensive systems. Measurements such as chest circumference, body length, and rump height can serve as practical predictors of body weight and productivity, particularly in resource-limited environments. The differentiation of ecotypes provides a functional basis for targeted breeding strategies, allowing selection of animals better adapted to specific production objectives, such as mobility, resilience, or meat yield.

A major strength of this study lies in its comprehensive approach, integrating morphological, morphometric, and multivariate analyses across multiple districts, thereby providing a robust morphostructural dataset for Creole goats in the Peruvian Amazon. The use of standardized protocols and PCA strengthens the reliability and interpretability of the findings.

However, certain limitations should be acknowledged. The study was based on cross-sectional data and did not incorporate longitudinal performance traits such as growth rate, reproductive efficiency, or milk yield. In addition, environmental and nutritional factors were not quantitatively modeled, and molecular analyses were not included to support the observed phenotypic variability.

Future research should focus on integrating morphometric data with genetic, reproductive, and productive performance indicators to develop comprehensive selection indices. Longitudinal studies evaluating growth, fertility, and adaptability under varying environmental conditions are needed. Additionally, incorporating molecular tools would enable validation of phenotypic patterns and support conservation strategies to preserve local genetic resources.

In conclusion, the study confirms that Creole goats in the Peruvian Amazonian exhibit significant phenotypic diversity and adaptive morphostructural traits shaped by environmental and management conditions. The combined use of body measurements, indices, and PCA provides a robust framework for characterization and selection, contributing to sustainable genetic improvement, conservation, and productivity enhancement in tropical goat production systems.

## DATA AVAILABILITY

The supplementary data can be made available from the corresponding author upon request.

## AUTHORS’ CONTRIBUTIONS

AR-V: Performed the data analysis and interpretation and contributed to manuscript drafting. LT-G: Contributed to the conceptualization and study design, as well as to the investigation and results visualization. EAS: Contributed to data analysis and interpretation and to manuscript review and editing, contributed to the investigation, translation, and manuscript drafting, as well as to its review and editing. JR-C: Contributed to the conceptualization of the study and participated in the drafting, review, and editing of the manuscript. CB-C: Contributed to manuscript review and editing. JC-L: Contributed to the study conceptualization, critical manuscript review, and overall supervision of the work. All authors have read and approved the final version of the manuscript.
